# The Natural Means of Preserving Health

**Published:** 1844-01

**Authors:** 


### THE NATURAL MEANS OF PRESERVING HEALTH.

We thank our friend, Dr. Deavenport, for a copy of a pamphlet with the above title, written by his neighbor, Dr. J. P. Epperson, of Pulaski, Tennessee. We can readily believe Dr. Deavenport's statement, that the author is a young man of acknowledged talents, and that his present effort is but an earnest of what he can and *will* do in his profession. It is long since we met with a pamphlet containing so much sound thought and good writing, from the pen of a young author. His subject is one which has been written upon by Johnson, by Combe, by Caldwell, and a host of strong men, but Dr. Epperson has treated it so well, that it assumes in his hands much of the freshness of a new topic. Such a writer may exercise a most healthful influence over the popular mind, for which his present tract was published. The truths he has therein told the people are of vital importance, and are communicated in language so simple and so natural that all can comprehend it, and, we should think, all must admire it. He treats of *exercise, the proper regulation of the mind, pure air, and aliment,* as the means of preserving health, and of eradicating the pre-disposition to consumption, and other hereditary diseases. The pamphlet ought to be extensively read by the people, and if its precepts are heeded, it will avert many an attack of dyspepsia and consumption. The following extracts, taken from different parts of the pamphlet, are but fair specimens of the pleasing manner in which the author conveys his practical truths:

"The first I shall notice, and that which is more frequently neglected than any other, is *Exercise.* If the question were asked, what is the most important rule of health, it might be answe.ed, as Demosthenes answered of oratory, "action! action!" It might as reasonably be expected that vegetation could spring forth, grow, and flourish without heat, light, or moisture, as that the animal frame could be fully developed, and preserved in health, without vigorous *action.* It is an indispensable means; it is indicated by the structure of the human frame; its long levers, and numerous and powerful muscles attached to them,—the arms and the legs, the hands and the feet, and the numerous joints, and their mobility, all obviously indicate that it is formed for vigorous, complicated, and repeated action.

"*Exercise* is an *instinctive* want in the animal economy. It may be seen in the aimless motions of infancy, in the romping of childhood and youth, and in the restlessness under restraint of more mature age. Where nature points out the rule of action, it cannot be disregarded with impunity. Even inanimate things are preserved by motion. The waters are purified by the agitation of streams, waves and tides. The atmosphere by its ever varying currents. Vegetation is continually waved by the veering winds, and is strengthened by their influence. But motion is more especially a function of animal beings, on the exercise of which the perfection of their growth, and their preservation most directly depends."

"Men are always seeking some universal principle, which they expect to do every thing: such as the perpetual motion in mechanics, a universal solvent in chemistry, a panacea in medicine, etc.; and contrary to all experience, and induction from established facts, they aie prone to disregard the fixed laws of nature, to run after such fantastical illusions. And of all illusions, that is the most fatal which leads us to overlook the *natural* actions of the system, and the *natural* circumstances by which they are promoted, and to vainly expect benefit from *medicine* in cases, in which, from the very nature of things, it must be inefficient, if not injurious. The influence of medicine on the system is a *borrowed* capital, which is necessary to extricate it from exigencies arising from neglect of the economy of health; but which, if resorted to frequently, must finally exhaust the estate it is intended to save. For medicine cannot create vital action, but can only modify it in a small degree, and its efficiency must be just in proporton to the degree of perfection of the *natural* actions of the bodily organs,—in other words, in proportion to the strength of the constitution. Therefore, to administer medicine with the view to improve the constitution, is to presuppose the existence of that which it is proposed to restore.

"I do not intend by this to disparage medical science; but to limit its application to is proper sphere. Most persons look on medicine as a perfect fountain of absolution, in which they can bathe and be absolved from all the diseases which their violation of the laws of health may incur.

"I do not maintain that human nature could be made exempt from disease and death, by any means. For all organized beings are necessarily subject to these.

"But my position is, that all diseases of the human system will be less frequent, and less severe, in proportion to its development, and maintainance of its organic powers, by the observance of the laws which regulate their actions. In the same manner, that crime will be prevented, in proportion to the judiciousness of legislation, and the excellency of moral precepts observed, although evil be a constituent part of human nature.

"2ndly. That constitutional diseases being the accumulated result of repeated *violations* of the laws of health, can be prevented by continued *observance* of these laws; and that their only cure, is their prevention. And although many diseases might have been placed in this class, I have particularized consumption only, on account of the frequency of its occurrence, and its fatality. And I have noticed dyspepsia in the same connection, because though not properly a constitutional disease, it is curable only by the same means.

"3dly. I advocate the doctrine that an ounce of prevention of disease by *natural means*, is better than a pound of cure by *medicine*. And yet I insist, on the *judicious* use of medical agents as a necessary evil in all diseases, whether unavoidable, or the result of imprudence. But at the same time I insist, that from the nature of the causes which produce consumption and dyspepsia, medicines can only act as palliatives in the treatment of them.

"4thly. And therefore I urge, that the public mind is too much fixed on medicine in the treatment of *these* diseases, to the neglect of the only possible means of curing them, by avoiding or correcting the causes which produce them.

"And these truths being settled, I base upon them the broad declaration, that whoever asserts that he can cure *any disease* by nostrums, or specifics, or by any mode of routine medication, without regard to the removal of its causes, and to the peculiarities of each particular case, is either grossly ignorant of the true principles of medical science, or is grossly attempting to impose on the credulity of the public; or, more likely both of these, for ignorance and imposition generally go together I make the declaration in this strong language, because I know, and deplore the greediness of the wonder-loving populace to swallow the boasted wonder-workings of quackery, and the restless eagerness of suffering and decaying humanity to grasp continually at the delusive shadows of hope, although constantly deceived by them. It does *seem* that men ought to 'learn wisdom by experience;' but verily, they 'wax worse and worse, deceiving and being deceived.'" Y.

